# Transcriptomics Analysis of the Adipogenic Differentiation Mechanism of Bovine Adipose-Derived Neural Crest Stem Cells

**DOI:** 10.3390/ani15162353

**Published:** 2025-08-11

**Authors:** Kai Zhang, Xiaopeng Tang, Rui Zhao, Yibo Yan, Xianyi Song

**Affiliations:** 1 College of Animal Science, Shanxi Agricultural University, Taiyuan 030032, China; zhangkai2255@163.com (K.Z.); zr734948896@163.com (R.Z.); 206434yyb@sxau.edu.cn (Y.Y.); 2 State Engineering Technology Institute for Karst Desertfication Control, School of Karst Science, Guizhou Normal University, Guiyang 550025, China

**Keywords:** adipogenic differentiation, bovine adipose-derived neural crest stem cells, differentially expressed genes, transcriptomics

## Abstract

The aim of the present study was to investigate the expression of related genes during the differentiation process of baNCSCs into adipocytes using transcriptomics technique, thereby clarifying the potential mechanism underlying baNCSCs differentiation into adipocytes and providing insights into lipid metabolism and regulation of lipid deposition in ruminants. Transcriptomic analysis was conducted on the adipocytes of baNCSCs on days 0 (CON0), 3 (DIF3), and 9 (DIF9) of differentiation. The results showed that in the early stage of adipocyte differentiation of baNCSCs, differentially expressed genes (DEGs) are mainly involved in metabolic pathways such as chromosome modification, cell cycle progression, and regulation of stem cell pluripotency. In the middle and late stages of differentiation, DEGs are mainly involved in metabolic pathways such as changes in cell morphology and synthesis of fatty acids and triglycerides. Predicting the top 10 core hub genes (CHGs) in the protein–protein interaction (PPI) network that regulate various differentiation stages of adipocytes reveals that *ERBB2*, *EGFR*, and *MYC* are upregulated during the early differentiation stage. In contrast, *ITGB1*, *KRAS*, *CCND1*, *ACTB*, *VEGFA*, *MET*, and *HRAS* are downregulated. During the middle and late stages of differentiation, the expressions of *TP53*, *CASP3*, *STAT3*, *CTNNB1*, *JUN*, *EGFR*, and *MYC* are upregulated, while *IGF1R*, *PTEN*, and *HRAS* are downregulated. In conclusion, the primary enrichment pathways of DEGs vary at distinct stages of adipocyte induction and differentiation in baNCSCs.

## Simple Summary

The use of bovine adipose-derived neural crest stem cells (baNCSCs) to study the adipogenic differentiation mechanism of bovine NCSCs can effectively avoid the species differences brought about by traditional cell line studies, such as those of humans and rodents. In the present study, transcriptomics was used to explore the expression of related genes during the adipogenic differentiation process of baNCSCs. The results indicated that the primary enrichment pathways of differentially expressed genes (DEGs) vary at distinct stages of adipocyte induction and differentiation in baNCSCs. This study initially revealed the mechanism of differentiation of bovine NCSCs into adipocytes, which can provide support for the study of lipid metabolism patterns and fat deposition regulation in ruminants.

## 1. Introduction

Adipocyte differentiation is a complex process, involving pluripotent stem cells or precursor cells being regulated by a series of transcriptional regulators under the influence of internal and external factors, and eventually transforming into mature lipid-filled adipocytes [1]. The core of this process lies in the timing expression of key genes such as peroxisome proliferator-activated receptor γ (PPARγ), fatty acid binding protein 4 (FABP4), CCAAT/enhancer binding protein α (C/EBPα), and adipose triacylglyceride lipase (ATGL), which regulates metabolic processes such as glycerol metabolism, fatty acid oxidation, and carbohydrate transformation in a network pattern [2,3,4]. The formation of adipocytes is closely related to the quality of animal meat [5]; therefore, studying the mechanism of adipocyte differentiation is of great economic significance for livestock and poultry breeding.

Neural crest stem cells (NCSCs) are progenitor cells with pluripotent differentiation potential, which can not only differentiate into neurons and glial cells but also into mesoderm and ectoderm derivatives and serve as transitional structures during early vertebrate embryonic development [6,7]. This multidirectional differentiation potential makes NCSCs an important candidate cell source for regenerative medicine and tissue engineering [8]. Adipogenesis is one of the differentiation pathways of NCSCs [9]. In particular, adipose-derived neural crest stem cells (AD-NCSCs) have been selected due to their neural crest origin, limited adipocyte differentiation, and greater experimental specificity, making them more suitable for targeted studies on fat formation than stromal vascular fraction cells or intramuscular adipose-derived stem cells [9,10]. Adipocyte differentiation involves various signaling pathways and regulatory factors, leading to changes in lipid profiles both on the cell surface and internally [11,12]. By tracking and comparing the alterations in cellular biomarkers at different stages of NCSCs differentiation, the biological processes (BPs) and stages of adipogenesis can be identified.

Jinnan cattle is one of the five major yellow cattle breeds, primarily distributed in the southern region of Shanxi Province, China. This breed serves not only as traditional draft cattle but also plays a significant role as an important beef cattle breed [13]. Currently, the breeding efforts for high-quality Jinnan cattle predominantly emphasize meat production. The quality of the meat, characterized by attributes such as marbling, tenderness, and flavor, has a direct impact on the market price of beef and consumer preferences [14]. By investigating and enhancing the meat quality of Jinnan cattle, we can increase their commercial value and foster the development of local animal husbandry. Fat plays a significant role in maintaining the life activities and metabolism of cattle [15]. Among them, the intramuscular fat content is closely related to the juiciness, palatability, and tenderness of the meat [16]. Therefore, analyzing the mechanism of adipogenic differentiation in beef cattle plays a guiding role in improving the quality of beef. The mouse C3H10T1/2 mesenchymal stem cells [17] and 3T3-L1 preadipocytes [18] have been commonly utilized as cell models for studying adipocyte differentiation. However, employing bovine-specific cells to investigate adipocyte differentiation may provide deeper insights into the mechanisms underlying bovine adipocyte differentiation. Currently, there have been reports on the mechanisms of the adipogenic differentiation of AD-NCSCs [9,10,19]. For instance, a study in mice adipose-derived stem/stromal cells (ASCs) has shown that neural crest-derived adipose stem cells express both neural crest markers (p75NTR, Nestin, Sox2) and preadipocyte/adipogenic markers (CD24, CD34, S100, Pref-1, GATA2, C/EBP-delta) [9]. A study in human ASCs showed that genes such as *CEBPA*, *PPARG*, *ADIPOQ*, and *aP2* are central to adipogenic differentiation, which facilitate the transition from stem cells to mature adipocytes and are frequently utilized as markers in transcriptomic studies [19]. However, research on the adipogenic differentiation mechanisms of bovine-derived cells is limited. For this reason, we isolated bovine adipose-derived neural crest stem cells (baNCSCs) from Jinnan cattle [20] to explore the mechanisms of the adipogenic differentiation in bovine NCSCs. Therefore, the aim of the present study was to investigate the expression of related genes during the differentiation process of baNCSCs into adipocytes using the transcriptomics technique, thereby clarifying the potential mechanism underlying baNCSCs differentiation into adipocytes in beef cattle, thereby offering a valuable reference for the meat quality control of Jinan cattle.

## 2. Materials and Methods

### 2.1. baNCSCs Isolation, Cell Culture, and Passage Procedure

Healthy male Chinese Jinan cattle (aged 6 months and weighing around 240 kg) were provided by the college of Animal Science, Shanxi Agricultural University (Taigu, China). The isolation and culture of baNCSCs were described in our previous study [20]. Briefly, the subcutaneous adipose tissues were obtained using minimally invasive ablation by biopsy from healthy Chinese Jinnan cattle. Under sterile conditions, the adipose tissues were repeatedly rinsed with PBS and subsequently transferred to a Petri dish containing a small amount of DMEM medium. The outer tissues, along with visible blood vessels and connective tissues, were removed using scissors, and the adipose tissue was then scraped into thin pieces with a scalpel, and placed in the growth medium [DMEM medium (high glucose, Glutamax; Gibco, Carlsbad, CA, USA) supplemented with 10% fetal bovine serum (FBS; Gibco, Carlsbad, CA, USA), 1 mM sodium pyruvate (Sigma, Saint Louis, MO, USA), 50 mg/mL uridine (Sigma, Saint Louis, MO, USA), 50 units/mL pen strep (Gibco, Carlsbad, CA, USA), and 1.25 mg/mL amphotericin B (Gibco, Carlsbad, CA, USA)], then subsequently explanted in wells pre-coated with collagens. Cells were dissociated from the wells with accutase (Sigma, Saint Louis, MO, USA) and seeded in normal culture dishes with the same medium at 37 °C in a 5% CO_2_ incubator and passaged with a ratio of 1:3 when cell density reaches to 70 to 80% confluence.

### 2.2. Transcriptome Detection of Adipocytes from baNCSCs at Different Differentiation Stages

The baNCSCs (2.1 × 10^4^/cm^2^) that had been passed down to 5 generations (P5) were seeded in a 10 cm culture dish (NEST, Wuxi, China), and cultured in the growth medium at 37 °C in a 5% CO_2_ incubator. Once the density of P6 generation cells in the culture dish reached 100% and after a period of 1 day without contact inhibition, adipocyte differentiation was induced using an adipogenic differentiation medium, which consisted of α-MEM medium (Gibco, Carlsbad, CA, USA) supplemented with 1% adipogenic supplement (R&D, Minneapolis, MN, USA), 10% FBS (Gibco, Carlsbad, CA, USA), 50 units/mL pen strep (Gibco, Carlsbad, CA, USA), and 1.25 mg/mL amphotericin B (Gibco, Carlsbad, CA, USA). The adipogenic differentiation medium was refreshed every 3 days. Cell samples were collected on the 0th day, the 3rd day, and the 9th day and were designated as the control group (CON0), the 3-day differentiation group (DIF3), and the 9-day differentiation group (DIF9), respectively. The experiments were conducted in triplicate.

The total RNA extraction and RNA-Seq were accomplished by Novogene (Beijing, China). Briefly, total cellular RNA was extracted using TRIzol reagent (Thermo, San Jose, CA, USA) in accordance with the manufacturer’s protocol. The concentration and purity of the total RNA were detected by NanoDrop-2000 (Thermo, San Jose, CA, USA). The integrity of the total RNA was determined by 1.5% agarose electrophoresis and an Agilent 2100 Bioanalyzer (Agilent Technologies, Santa Clara, USA). Qualified RNA samples were subsequently used for cDNA libraries construction.

The cDNA libraries were subjected to high-throughput sequencing using the Illumina HiSeq 2500 platform to generate 150 bp paired-end reads. The raw data was processed to remove adapter sequences, poly-N or adaptors, and low-quality reads. Further quality filtering was conducted to obtain clean reads. The clean reads were aligned to the bovine genome sequence database (Bos taurus UMD 3.1.1) available on the NCBI website using HISAT2 software (v2.2.1), followed by genomic annotation of the matched sequences. The fragments per kilobase of transcript per million mapped reads (FPKM) values of all genes between groups were compared using DESeq2’s model (version 1.30.1), which is based on the negative binomial distribution. DIF3 and DIF9 were compared with CON0, denoted as DIF3 vs. CON0 and DIF9 vs. CON0, respectively. Genes with |log2(FC)| ≥ 1 and FDR < 0.05 were identified as differentially expressed genes (DEGs) based on corrected *p* values using the Benjamini–Hochberg and Hochberg methods. DEGs were subjected to gene ontology (GO) functional classification and the Kyoto Encyclopedia of Genes and Genomes (KEGG) signaling pathway enrichment analysis using ClusterProfiler software (Version 4.17.0). A *p* value < 0.05 was considered statistically significant.

The DEGs identified in the comparisons of DIF3 vs. CON0 and DIF9 vs. CON0 were entered into the online STRING database to construct a protein–protein interaction (PPI) network with a confidence threshold of 0.4. The network visualization was performed in Cytoscape (version 3.9.1). The top 10 core hub genes (CHGs) in the PPI network were predicted using the Maximum group centrality (MCC) analysis algorithm of CytoHubba.

The sequencing results were validated by randomly selecting 10 genes from DEGs. Among them, fatty acid binding protein 4 (*FABP4*), peroxisome proliferators-activated receptors γ (*PPARγ*), adipocyte enhancer binding protein 1 (*AEBP1*), ATP-binding aassette subfamily A member 10 (*ABCA10*), and adiponectin receptor 2 (*ADIPOR2*) were identified as DEGs in the comparison of DIF3 vs. CON0. Conversely, Kruppel-like factors (*KLF4*), stearoyl-CoA desaturase 1 (*SCD1*), CD36 antigen (*CD36*), insulin-induced gene 1 (*INSIG1*), and uncoupling protein 2*UCP2* were identified as DEGs in the comparison of DIF9 vs. CON0. The gene expression levels were determined using quantitative PCR (qPCR) to validate the reliability of the transcriptome sequencing results. The experimental procedures for total RNA extraction, detection, reverse transcription, and cell reaction system were consistent with our previous study [20]. The primer sequences used are provided in Table 1.

## 3. Results

### 3.1. RNA-seq Quality Control and Reference Genome Alignment Results

The quality control of transcriptome sequencing data is shown in Table 2. The number of filtered reads obtained for each group of samples ranges from 39,759,198 to 50,051,578. The percentage of bases with a quality score of ≥30 in the total bases of the samples is 94.53% to 95.51%. The distribution of GC base content in the samples conforms to the base composition rule, ranging from 44.96% to 52.34%, indicating high-quality sequencing data. The alignment efficiency range of clean reads generated by the samples with the reference genome sequences was 90.67–94.43%, and the proportion of reads that could specifically align to the bovine reference genome was 88.76–92.20%, indicating relatively high alignment efficiency, coverage rate, and reliability of the transcriptome results.

**Figure 1:**
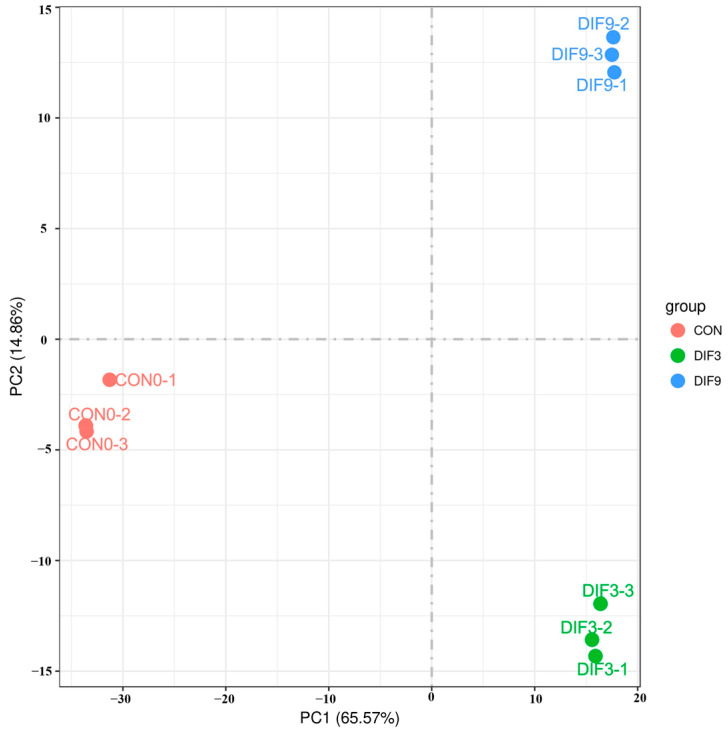
Principal component analysis. Cell samples were collected on the 0th day, the 3rd day, and the 9th day and designated as the control group (CON0), the 3-day differentiation group (DIF3), and the 9-day differentiation group (DIF9).

Principal component analysis (PCA) was performed on the FPKM of all samples from the CON0, DIF3, and DIF9 groups (Figure 1). The results indicated a clear separation of sample points into distinct groups, with samples within each group clustering closely together. Furthermore, the samples from different groups exhibited significant discrimination, suggesting high repeatability within groups and notable differences among samples from varying groups.

### 3.2. Analysis of Differentially Expressed Genes

**Figure 2:**
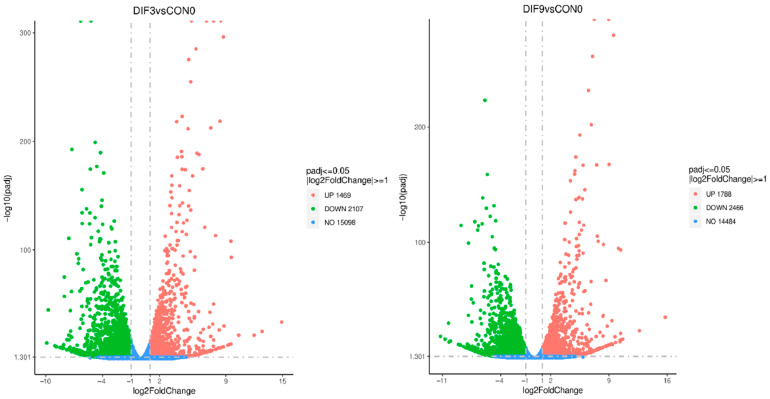
Volcanic map of differentially expressed genes at different stages of adipocyte differentiation. The horizontal coordinate is the change of gene expression factor between the two groups, the vertical coordinate is the significance level of gene expression difference between the two groups, and the dashed blue line is the threshold line of screening criteria for differential genes.

Genes within the threshold were classified as DEGs based on the screening criteria of |log2(FC)| ≥ 1 and FDR < 0.05 (Figure 2). It showed that a total of 3576 DEGs were screened out from DIF3 vs. CON0, among which 1469 genes were upregulated and 2107 genes were downregulated. A total of 4254 DEGs were screened out from DIF9 vs. CON0, among which 1788 genes were upregulated and 2466 genes were downregulated.

### 3.3. Gene Ontology Functional Enrichment Analysis of Differentially Expressed Genes

**Figure 3:**
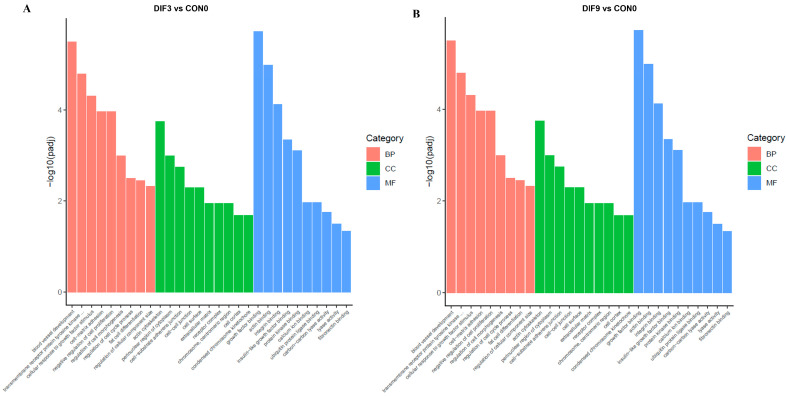
GO enrichment analysis of differentially expressed genes at different stages of adipoblastic differentiation. (**A**) DIF3 vs. CON0; (**B**) DIF9 vs. CON0. BP: biological processes; CC: cellular components; MF: molecular functions. The y-coordinate is the significance level of GO term enrichment, and the x-coordinate is the name of GO term.

To elucidate the biological functions of DEGs during adipocyte differentiation, GO functional enrichment analysis was separately performed for DEGs in DIF3 vs. CON0, as well as in DIF9 vs. CON0 (Figure 3). It showed that DEGs were significantly enriched in 447 functional categories in the early stage and 358 functional categories in the middle to late stages of differentiation. These DEGs were categorized into three major groups: BP, cellular components (CC), and molecular functions (MF). The top 10 most significant terms for each major category were selected to create bar charts. The DEGs of DIF3 vs. CON0 are mainly enriched in GO terms related to vascular development, response to biological stimuli, cell morphology involved in differentiation, cell cycle process, and adipocyte differentiation in the BP. In terms of CC category, they are enriched in locations such as the cell surface, nucleus vicinity, chromosomal part, and extracellular space. Within the MF category, GO terms like protein kinase binding, calcium ion binding, and insulin-like growth factor binding were enriched (Figure 3A). For the DIF9 vs. CON0, DEGs of are mainly enriched in GO terms such as regulation of vascular development, response to organic nitrogen compounds, negative regulation of catalytic activity, and regulation of mitogen-activated protein kinase (MAPK) cascades in BP category; GO terms such as cell surface cytoskeleton, extracellular matrix, and complex receptors in CC category; and GO terms such as receptor binding and regulation of MAPK tyrosine/threonine/serine phosphatase activity in the MF category (Figure 3B).

### 3.4. Enrichment Analysis of KEGG Pathways for Differentially Expressed Genes

**Figure 4:**
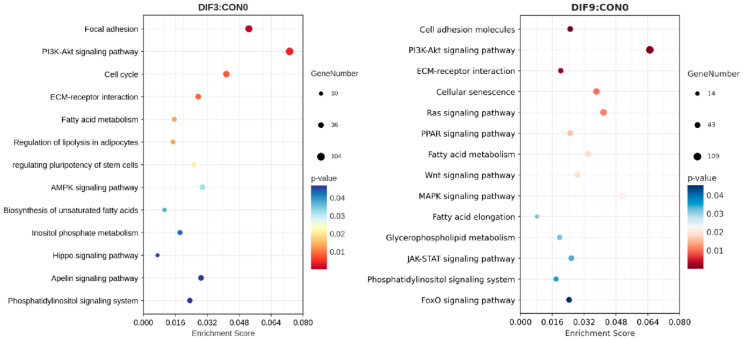
KEGG pathway scatter plot of DEGs at different stages of adipocyte differentiation. The x-coordinate is the ratio of the number of differential genes enriched in this pathway to the total number of differential genes, and the y-coordinate is the name of the KEGG enrichment pathway.

To clarify the regulatory role of DEGs in adipocyte differentiation and the associated signaling pathways, KEGG pathway enrichment analysis was performed on DEGs from DIF3 vs. CON0, as well as DIF9 vs. CON0 (Figure 4). The results indicated that DEGs from DIF3 vs. CON0 are co-enriched in 315 pathways, with significant enrichment in 77 pathways, including pathways such as phosphoinositide 3-kinase-serine/threonine kinase (PI3K-AKT), cell cycle, ECM–receptor interactions, and fatty acid metabolism. Similarly, DEGs from DIF9 vs. CON0 were enriched in 316 pathways, with significant enrichment in 85 pathways, including metabolic pathways like PI3K-AKT, PPAR, forkhead box O (FoxO), MAPK, fatty acid metabolism, and glycerophospholipid metabolism.

### 3.5. Screening of Core Hub Genes in Adipogenic Metabolism

**Figure 5:**
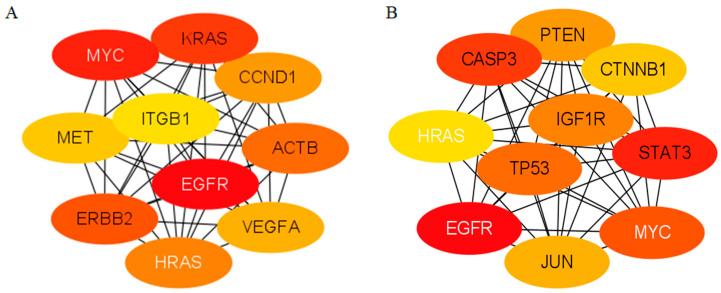
Screening of core hub genes involved in adipogenic metabolism. (**A**) DIF3 vs. CON0; (**B**) DIF9 vs. CON0. The white annotated genes are the common core hub gene. The hub color from dark to light indicates the assignment from high to low. ACTB: β-actin; CASP3: caspase 3; CCND1: cyclin D1; CTNNB1: catenin beta 1; EGFR: epidermal growth factor receptor; ERBB2: Erb-b2 receptor tyrosine kinase 2; HRAS: HRas proto-oncogene; IGF1R: insulin-like growth factor 1 receptor; ITGB1: integrin subunit beta 1; JUN: JUN proto-oncogene; KRAS: KRAS proto-oncogene; MET: receptor tyrosine kinase; MYC: MYC proto-oncogene; PTEN: phosphatase and tensin homolog; TP53: tumor protein p53; STAT3: signal transducer and activator of transcription 3; VEGFA: vascular endothelial growth factor A.

The expression levels of DEGs were integrated with the protein–protein interaction network to screen the core hub genes involved in adipogenic metabolism (Figure 5). Eight adipocyte metabolic signaling pathways with significant enrichment of DIF3 vs. CON0 were identified: PI3K-AKT, fatty acid metabolism, Hippo, biosynthesis of unsaturated fatty acids, apelin, regulation of adipocyte lipolysis, phosphatidylinositol signaling system, and inositol phosphate metabolic signaling pathway. DEGs in these pathways were analyzed for protein network interactions using the STRING online database and Cytoscape software. The top 10 CHGs identified were *MYC*, *KRAS*, *CCND1*, *ACTB*, *VEGFA*, *HRAS*, *ERBB2*, *MET*, *ITGB1*, and *EGFR* (Figure 5A). Similarly, eight adipocyte metabolic signaling pathways with significant enrichment of DIF9 vs. CON0 were identified: PI3K-AKT, FoxO, Ras, MAPK, fatty acid metabolism, Wnt, glycerophospholipid metabolism, and PPAR signaling pathways. The top 10 CHGs identified are *IGF1R*, *HRAS*, *PTEN*, *TP53*, *STAT3*, *MYC*, *JUN*, *CASP3*, *CTNNB1*, and *EGFR* (Figure 5B). The common core hub genes present in both stages of cell differentiation are *HRAS*, *EGFR*, and *MYC*.

As shown in Table 3, in the early stage of cell differentiation (DIF3 vs. CON0), the genes upregulated are *ERBB2*, *EGFR*, and *MYC*, while the downregulated genes are *ITGB1*, *KRAS*, *CCND1*, *ACTB*, *VEGFA*, *MET*, and *HRAS*. In the middle and late stages of differentiation, the genes showing upregulated expression include *TP53*, *CASP3*, *STAT3*, *CTNNB1*, *JUN*, *EGFR*, and *MYC*, whereas the downregulated genes are *IGF1R*, *PTEN*, and *HRAS*. The changing trends of the core hub genes *HRAS*, *EGFR*, and *MYC* are consistent throughout the two stages of differentiation.

### 3.6. Quantitative Verification of Differentially Expressed Genes in the Transcriptome

**Figure 6:**
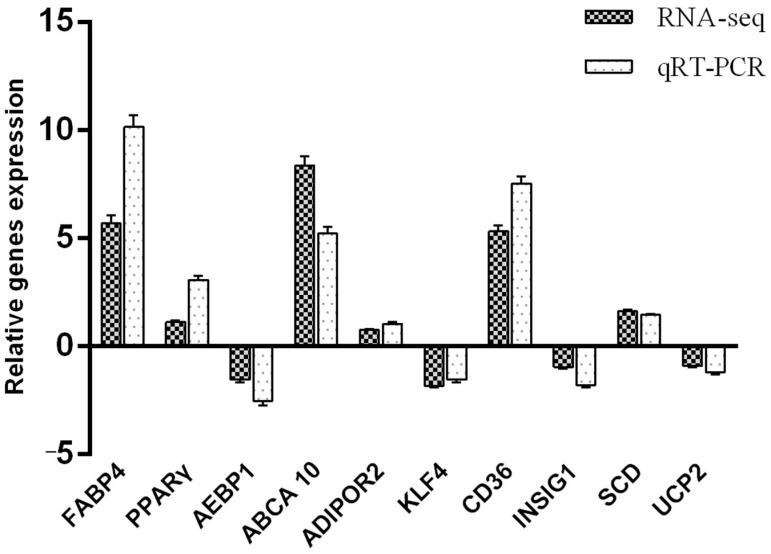
Comparison of RNA-seq and qPCR results. ABCA10: ATP-binding aassette subfamily A member 10; ADIPOR2: adiponectin receptor 2; AEBP1: adipocyte enhancer binding protein 1; CD36: CD36 antigen; FABP4: fatty acid binding protein 4; INSIG1: insulin-induced gene 1; KLF4: Kruppel-like factors; PPARγ: peroxisome proliferators-activated receptors γ; SCD1: stearoyl-CoA desaturase 1; UCP2: uncoupling protein 2.

The *FABP4*, *PPARγ*, *AEBP1*, *ABCA10*, and *ADIPOR2* genes from the DIF3 and CON0 groups, and *KLF4*, *SCD1*, *CD36*, *INSIG1*, and *UCP2* genes from the DIF9 and CON0 groups were randomly selected to be detected by qRT-PCR to verify the transcriptome sequencing results (Figure 6). The results showed that the transcriptome sequencing data is consistent with the expression patterns of the corresponding RNA-Seq genes, indicating the accuracy and reliability of the transcriptome sequencing results.

## 4. Discussion

Studying gene expression and transcript structure at specific developmental stages using transcriptomic methods is crucial for understanding organism development and metabolic regulation. To elucidate the molecular mechanisms underlying the differentiation of baNCSCs into mature adipocytes, mRNA from baNCSCs at various differentiation time points was extracted for transcriptome sequencing analysis to identify the role of DEGs and signaling pathways in regulating adipocyte differentiation and formation.

Through GO functional annotation and enrichment analysis, it was found that during the early stage of differentiation, DEGs were mainly enriched in GO terms related to BP, such as the cell cycle process and the morphology of differentiated cells; CC, such as perinuclear and chromosomal parts; and MF, including receptor binding. In the middle and late stages of differentiation, DEGs are mainly distributed in GO terms, such as the negative regulation of catalytic activity in BP, the regulation of MAPK cascade, and the regulation of the cell surface skeleton, extracellular matrix in CC, as well as the regulation of enzymes activities in MF. The results of GO terms suggest a correlation between chromatin epigenetic modifications and cell differentiation, indicating that changes in chromosomes during the early stages of differentiation are essential for cells to fully realize their differentiation potential. The adipogenic differentiation of NCSCs consists of the permission stage during the contact inhibition period and the executive stage, which is independent of the cell cycle [21]. Once the cells undergo contact inhibition and are exposed to inducing factors, they progress into the executive stage of differentiation, resulting in the re-entry of cells into the cell cycle, an increase in cell number, and a gradual morphology transition from fibrous to round. Alterations in cell cycle regulatory factors, cell metabolic patterns, and cell signal transduction pathways, along with the activities of chromatin epigenetic modification factors and alterations in gene sequence methylation status, establish chromatin epigenetic rules that regulate adipocyte differentiation potential [22,23]. Signal transduction is an important function of cell membrane proteins and can activate the signal pathways related to adipogenic differentiation of NCSCs [24,25]. The results of this study corroborate this perspective, demonstrating that in the early stage of differentiation, a significant enrichment of DEGs related to signal transduction, such as protein kinase binding, calcium ion binding and insulin-like growth factor binding, was achieved. During the middle and late stages of differentiation, the types of cytoskeletal components and extracellular matrix components change [26]. Cells evolve from spindle-shaped to nearly round, and the expression of actin and tubulin, the key components of the eukaryotic cytoskeleton, decreases to regulate the structure and dynamic functions of cytoskeletal elements and regulate basic biological processes such as lipogenesis and differentiation dependent on the cytoskeleton [27,28]. During the late stage of differentiation, immature adipose-derived stem cells are embedded in the extracellular matrix, where differentiation-inducing signaling factors regulate the activation or inhibition of adipogenic enzymes by binding to cell surface receptors, accompanying processes such as fatty acid elongation, fatty acid metabolism, and lipid synthesis, leading to the accumulation of lipid droplets and the eventual formation of mature adipocytes [29,30].

Using the KEGG database for pathway enrichment analysis of DEGs, it was found that during the early differentiation stage, DEGs related to lipid metabolism regulation were significantly enriched in the PI3K-AKT and cell cycle pathways. PI3K-AKT has been confirmed as a classic signaling pathway for fat development [31], and cell cycle regulation is closely related to fat deposition [32]. The PI3K-AKT pathway integrates signals that coordinate both cell proliferation and differentiation, ensuring that preadipocytes exit the cell cycle at the appropriate stage to become mature adipocytes [33,34]. In the middle to late stages of differentiation, DEGs were mainly enriched in the PPAR, MAPK, and glycerophospholipid metabolism pathways. These pathways are intimately associated with fatty acid and steroid metabolism, as well as adipogenesis [35]. PPARγ is the central transcription factor driving adipocyte differentiation, with its activation leading to increased expression of adipogenic markers and lipid accumulation [36]. MAPK signaling regulates the timing and extent of adipogenesis by phosphorylating key transcription factors, such as C/EBPβ and PPARγ, and can either promote or inhibit adipocyte formation depending on the context [37]. Glycerophospholipid metabolism is upregulated to support the increased demand for membrane and storage lipids as preadipocytes mature into adipocytes [36]. The present study is consistent with Huang et al. [38], who reported that DEGs in subcutaneous adipose tissue of Japanese Wagyu and Holstein cattle were enriched in biological processes and pathways related to fat synthesis and lipid metabolism, involving genes such as *PPARγ*, *PLIN2*, and *ELOVL6*.

The adipocyte differentiation of baNCSCs is regulated by multiple transcription factors, genes, and signaling pathways, with the gene regulatory network being the focus of adipocyte differentiation research. In the present study, the CHGs that regulate the differentiation of baNCSCs in the early stage are *ITGB1*, *KRAS*, *CCND1*, *ACTB*, *VEGFA*, *MET*, *HRAS*, *ERBB2*, *EGFR*, and *MYC*. These selected CHGs are all targeted genes related to regulating lipid metabolism, suggesting a potential link between adipocyte differentiation and cell migration, tumor growth, invasion, and metastasis. Therefore, they can serve as candidate genes influencing the adipogenic differentiation of baNCSCs, providing potential genetic markers for regulating fat deposition in bovine organisms from a molecular biology perspective. However, another study has found through the integration of genomics and transcriptomics techniques that the candidate genes affecting subcutaneous fat deposition in beef cattle are *ACACA*, *SCD*, *FASN*, *ACOX1*, *ELOVL5*, *HACD2*, and *HSD17B12* [39]. The differences in these results may be attributed to variations in experimental purposes and techniques. The CHGs identified in this study are involved in the upstream or midstream interactions within pathways associated with cell cycle regulation, adipogenesis, and lipid metabolism, while genes such as *ACACA*, *SCD*, and *FASN* play direct roles in fatty acid synthesis, oxidation, and metabolism, as well as in adipogenesis and glycolysis processes [40]. In addition, the present study found that there were differences in the types and expression levels of DEGs and CHGs between the early stage of differentiation and the middle and late stages of differentiation, indicating that core hub genes may interact and jointly participate in the signaling pathways related to the cell cycle and lipid metabolism, regulating the processes of lipid synthesis, transformation and degradation, and affecting the adipocyte differentiation of bovine baNCSCs.

*ERBB2*, *EGFR*, and *MYC* are genes that are upregulated in the early stage of adipocyte differentiation, contributing to the enhancement of adipocyte differentiation. ERBB2 and EGFR belong to the epidermal growth factor receptor family, forming heterodimers that regulate the proliferation, differentiation, and apoptosis of stem cells through signaling pathways such as PI3K-AKT, JAK-STAT, and MAPK [41]. Therefore, upregulation of their expression can enhance adipocyte differentiation capability. The present study identified 159, 122, and 56 DEGs enriched in the PI3K-AKT, JAK-STAT, and MAPK signaling pathways, respectively, during the early stage of differentiation, from which it can be inferred that heterodimers regulate adipocyte differentiation, lipid metabolism, and deposition by interacting with genes associated with these signaling pathways. The MYC gene is an oncogene that encodes nuclear proteins, including C-myc, N-myc, L-myc, and R-myc. The encoded protein is an intracellular DNA-binding protein involved in cell cycle regulation, which participates in the transcriptional activation of target genes and plays a crucial role in regulating cell differentiation and proliferation [42]. In addition, MYC can induce the expression of SREBP1, which together activate fatty acid synthesis, promoting the elongation of fatty acid chains using glucose and glutamine [43].

Downregulated genes during early differentiation include *ITGB1*, *KRAS*, *CCND1*, *ACTB*, *VEGFA*, *MET*, and *HRAS*, which can enhance adipocyte differentiation. ITGB1 is a conformed laminin cell surface receptor and a typical marker of cell adhesion, which can activate the differentiation potential of hematopoietic stem cells through the ITGB1–ILK–β catenin–JUN axis [44], and its decreased expression can affect the generation of skeletal muscle progenitor cells into adipocytes [45]. KRAS and HRAS are members of the RAS proto-oncogene family, which encode K-Ras and H-Ras proteins located on the inner side of the cell membrane, respectively. These proteins possess guanosine triphosphatase activity; interact with Raf kinase; participate in cell signal transduction; regulate downstream MAPK, PI3K, and phospholipase C pathways; and serve as crucial molecules in controlling cell proliferation, apoptosis, and differentiation [46,47]. CCND1 is a factor associated with the cell cycle signaling pathway that forms a complex with CDK4/6 to phosphorylate Rb, thereby activating the transcription factor E2F, which regulates the transition of the cell cycle from the pre-DNA synthesis stage to the DNA replication stage [48]. Therefore, the decreased expression of CCND1 may provide conditions for baNCSCs to maintain cell differentiation. ACTB (β-actin), as the primary component of cytoskeletal microfilaments, plays a crucial role as an important skeletal protein. A key indicator of cell differentiation is the alteration in cell morphology. The cytoskeleton facilitates intracellular signal transduction by interacting with the extracellular matrix, while stabilized actin plays a role in inhibiting adipocyte differentiation [49]. VEGFA is a growth factor that regulates vascular permeability and possesses functions such as inducing endothelial cell proliferation, promoting cell migration, and inhibiting cell apoptosis [50]. The overexpression of VEGFA can facilitate the browning of white adipose tissue, upregulate the expression of UCP1, and enhance thermogenesis. In adipose tissue, the short-term induction of VEGFA is associated with weight loss and a reduction in fat mass [51]. MET is a receptor tyrosine kinase that encodes the transmembrane receptor for hepatocyte growth factor and is involved in cell survival, migration, and proliferation through the PI3K-AKT-mTOR, MAPK-ERK, and Wnt/β-catenin signaling pathways [52,53].

The genes exhibiting upregulated expression during the middle and late stages of differentiation include *TP53*, *CASP3*, *STAT3*, *CTNNB1*, *JUN*, *EGFR*, and *MYC*. The changes of these genes promote cell differentiation and apoptosis of baNCSCs. TP53 encodes a tumor suppressor protein that induces cell cycle arrest and promotes apoptosis, thereby exerting its tumor suppressor effect [54]. CASP3 is also a cysteine protease related to apoptosis [55]. STAT3 is a crucial signal transducer in the JAK/STAT3 pathway. Upon activation by JAK, phosphorylation occurs at the Ser727 or Tyr705 sites, allowing STAT3 to translocate into the nucleus, where it binds to the promoter region of target genes to regulate their expression, playing a vital role in cell growth, differentiation, and apoptosis [56]. Moreover, during the process of adipogenic differentiation, the activation of the JAK/STAT3 signaling pathway can promote the formation of adipocytes [57]. CTNNB1 encodes the adhesive protein β-catenin, a core component of the classical Wnt signaling pathway, which is crucial for adipocyte differentiation as a transcriptional activator [58]. JUN encodes the c-Jun protein, which, in conjunction with the c-Fos protein, forms the transcription factor AP-1 characterized by a leucine zipper structure and is involved in regulating various processes, including cell proliferation, differentiation, immune responses, and inflammation [59].

The genes that exhibit downregulated expression during the middle and late stages of differentiation include *IGF1R*, *PTEN*, and *HRAS*, suggesting that the PI3K-AKT pathway serves as the primary regulatory mechanism. Consequently, the ability of cells to differentiate and accumulate lipids decreases during these stages. IGF1R is a cell surface receptor belonging to the tyrosine kinase receptor family and serves as a crucial component of the IGF1 signaling system. It modulates cell growth and differentiation through the PI3K-AKT, FoxO, Ras, and MAPK signaling pathways, thereby influencing lipid metabolism and the development of adipose tissue [60]. PTEN can convert PIP3 to PIP2 through dephosphorylation, which reduces AKT activation and inhibits the formation of stem cells into adipocytes, consequently limiting lipid accumulation [61].

## 5. Conclusions

In summary, in the early stages of adipocyte differentiation in baNCSCs, the DEGs are primarily involved in metabolic pathways related to chromatin modification, cell cycle progression, and the regulation of stem cell pluripotency. During the middle and late stages of differentiation, these genes predominantly participate in metabolic pathways associated with changes in cell morphology as well as the synthesis of fatty acids and triglycerides. The top 10 CHGs in the early stage of differentiation are *MYC*, *KRAS*, *CCND1*, *ACTB*, *VEGFA*, *HRAS*, *ERBB2*, *MET*, *ITGB1,* and *EGFR*. In the middle and late stages of differentiation, the CHGs are *IGF1R*, *HRAS*, *PTEN*, *TP53*, *STAT3, MYC*, *JUN*, *CASP3*, *CTNNB1* and *EGFR*. The common CHGs in the early stage of differentiation and the late stages of differentiation are *HRAS*, *EGFR,* and *MYC*. In conclusion, the primary enrichment pathways of DEGs vary at distinct stages of adipocyte induction and differentiation in baNCSCs.

## Figures and Tables

**Table 1:** The primer sequences of gene.

Gene	Primer Sequence (5′-3′)	Product Length (bp)	Accession No.
*FABP4*	forward: AGGTACCTGGAAACTTGTCTCCA	92	NM_174314.2
	reverse: CCATGCCAGCCACTTTCCTG		
*PPARγ*	forward: AGTGGAGCCTGTATCCCCAC	125	NM_181024.2
	reverse: ACCCTGACGCTTTATCCCCA		
*ADIPOR2*	forward: CCCGGCAAGTGTGACATCT	92	NM_001040499.2
	reverse: TTCGAGACCCCGTGGAAGT		
*CD36*	forward: GCATTCTGAAAGTGCGTTGA	179	NM_001278621.1
	reverse: CGGGTCTGATGAAAGTGGTT		
*SCD1*	forward: TTATTCCGTTATGCCCTTGG	151	OP920982.1
	reverse: GGTAGTTGTGGAAGCCCTCA		
*ABCA10*	forward: CGCCCAAGAAACGACTC	193	XM_070774182.1
	reverse: GAAAAGCCACAAACCCG		
*AEBP1*	forward: GGAGTGGGCTCCAGTAGAGA	175	XM_024991120.2
	reverse: CACGCCCCATCGTAGTAGTC		
*INSIG1*	forward: AGAGCCACACAAGTTCAAGC	288	NM_001077909.1
	reverse: AGCCAGGAGCGGATGTAGAG		
*KLF4*	forward: GGAGACGGAGGAGTTCAATGAT	118	XM_005210496.5
	reverse: GGACGAGGATGAGGCTGATG		
*UCP2*	forward: GTTCTACACCAAGGGCTCTGA	117	NM_001033611.2
	reverse: AACCGGACCTTCACCACAT		
*GAPDH*	forward: TGAACCACGAGAAGTATAACAACAC	125	NM_001034034.2
	reverse: GGTCATAAGTCCCTCCACGAT		

FABP4: fatty acid binding protein 4; PPARα: peroxisome proliferators-activated receptors α; ADIPOR2: adiponectin receptor 2; CD36: CD36 antigen; SCD1: stearoyl-CoA desaturase 1; ABCA10: ATP-binding aassette subfamily A member 10; AEBP1: adipocyte enhancer binding protein 1; INSIG1: insulin-induced gene 1; KLF4: Kruppel-like factors; UCP2: uncoupling protein 2; GAPDH: glyceraldehyde-3-phosphate dehydrogenase.

**Table 2:** Statistics of sample sequencing quality and reference genome comparison.

Sample	Clean Reads	Q30 (%)	GC Content (%)	Total Map (%)	Unique Map (%)
CON0-1	42,247,574	95.33	47.60	93.71	91.44
CON0-2	44,361,380	95.51	52.19	94.36	92.14
CON0-3	45,886,196	95.37	52.34	94.43	92.20
DIF3-1	50,051,578	95.43	50.87	92.42	90.47
DIF3-2	41,690,784	95.04	49.29	91.24	89.37
DIF3-3	41,965,278	94.53	49.37	90.67	88.76
DIF9-1	43,073,620	95.43	49.47	92.33	90.37
DIF9-2	41,494,356	95.14	45.55	93.64	91.70
DIF9-3	39,759,198	95.24	44.96	94.05	92.01

Clean reads are the number of clean reads after filtering; Q30 refers to the percentage of bases with Phred values greater than 30 in total bases; GC content is the percentage of G and C in the four bases; total map is the percentage of reads matched to the genome; unique map is the percentage of reads matched to the unique location of the reference genome.

**Table 3:** Expression of lipid metabolism core hub gene.

Genes	DIF3 vs. CON0	DIF9 vs. CON0	Significantly Enriched Lipid Metabolism Pathways
*ITGB1*	down	normal	PI3K-AKT signaling pathway
*KRAS*	down	normal	PI3K-AKT and apelin signaling pathway
*CCND1*	down	down	PI3K-AKT, hippo and apelin signaling pathway
*ACTB*	down	down	Hippo signaling pathway
*VEGFA*	down	down	PI3K-AKT signaling pathway
*MET*	down	down	PI3K-AKT signaling pathway
*ERBB2*	up	normal	PI3K-AKT signaling pathway
*HRAS*	down	down	PI3K-AKT, Apelin, FoxO, Ras, and MAPK signaling pathway
*EGFR*	up	up	PI3K-AKT, FoxO, Ras, and MAPK signaling pathway
*MYC*	up	up	PI3K-AKT, Hippo, MAPK, and Wnt signaling pathway
*IGF1R*	down	down	PI3K-AKT, FoxO, Ras, and MAPK signaling pathway
*PTEN*	normal	down	PI3K-AKT and FoxO signaling pathway
*TP53*	up	up	PI3K-AKT, MAPK, and Wnt signaling pathway
*STAT3*	up	up	FoxO signaling pathway
*JUN*	up	up	Wnt and MAPK signaling pathway
*CASP3*	up	up	MAPK signaling pathway
*CTNNB1*	normal	up	Wnt signaling pathway

ACTB: β-actin; AKT: serine/threonine kinase; CASP3: caspase 3; CCND1: cyclin D1; CTNNB1: catenin beta 1; EGFR: epidermal growth factor receptor; ERBB2: Erb-b2 receptor tyrosine kinase 2; FoxO: forkhead box O; HRAS: HRas proto-oncogene; IGF1R: insulin-like growth factor 1 receptor; ITGB1: integrin subunit beta 1; JUN: JUN proto-oncogene; KRAS: KRAS proto-oncogene; MAPK: mitogen-activated protein kinase; MET: receptor tyrosine kinase; MYC: MYC proto-oncogene; PI3K: phosphoinositide 3-kinase; PTEN: phosphatase and tensin homolog; TP53: tumor protein p53; STAT3: signal transducer and activator of transcription 3; VEGFA: vascular endothelial growth factor A.

## Data Availability

The data presented in this study is available on request from the corresponding authors.

## Abbreviations

The following abbreviations are used in this manuscript:

AKT	serine/threonine kinase
baNCSC	bovine adipose-derived neural crest stem cells
BP	biological processes
CC	cellular components
C/EBPα	CCAAT/enhancer binding protein α
CHGs	core hub genes
DEGs	differentially expressed genes
FABP4	fatty acid binding protein 4
FBS	fetal bovine serum
FC	fold change
FoxO	forkhead box O
FPKM	fragments per kilobase of transcript per million mapped reads
GO	gene ontology
KEGG	Kyoto Encyclopedia of Genes and Genomes
MAPK	mitogen-activated protein kinase
MCC	maximum group centrality
MF	molecular functions
PI3K	phosphoinositide 3-kinase
PPARγ	peroxisome proliferator-activated receptor γ
PPI	protein–protein interaction
